# How come Denmark is planning to increase the annual budget for psychiatry by almost 20 percent?

**DOI:** 10.1192/j.eurpsy.2023.2409

**Published:** 2023-08-16

**Authors:** Merete Nordentoft, Mikkel Rasmussen, Lene Høgh, Christian Legind, Jakob Kjellberg

**Affiliations:** 1 Mental Health Centre Copenhagen, Mental Health Services in the Capital Region of Denmark, Hellerup, Denmark; 2 Department of Clinical Medicine, Faculty of Health and Medical Science, University of Copenhagen, Copenhagen, Denmark; 3 Danish Psychiatric Society, Copenhagen, Denmark; 4 Central Denmark Region, Aarhus, Denmark; 5 Mental Health Centre Horsens, Mental Health Services in Central Region, Horsens, Denmark; 6 VIVE – The Danish Centre for Social Science Research, Copenhagen, Denmark

**Keywords:** Psychiatry, public health, burden of disease, mental health services

## Abstract

In Denmark, a 10-year plan for psychiatry has been agreed on. The content of the plan was developed in collaboration between the Danish Health Authority and the Danish Authority for Social Services and Housing, and it involved many stakeholders. Recently, the government presented a planned investment that would increase the overall budget in Danish regions and municipalities by almost 20 percent over a 10-year period. Epidemiological research demonstrating shortened life expectancy and high levels of burden of disease for people with mental disorders contributed to emphasizing the need for improvement of psychiatric services. User organizations, trade unions, and scientific societies in the field of mental health were unified in a common organization, called the Psychiatry Alliance, and this alliance agreed on common action points and acted together to influence politicians. An assertive approach toward politicians and media was pivotal, and being a first mover and presenting tentative budgets was very influential.

## Organization of Danish healthcare

Psychiatric treatment in Denmark is organized through a public healthcare system, that is publicly funded and covers the whole population [1]. It is divided into the primary and the secondary healthcare sector. In the primary healthcare sector, all citizens have their own general practitioner, who can provide basic treatment and refer them to private specialists, to social services, or to hospital-based in- and out-patient facilities in the secondary healthcare sector. Secondary healthcare is organized in five Danish regions. Since 2013, psychiatric disorders, with at least moderate severity, are covered by the “right to evaluation and treatment” in the secondary health sector within 30 days. This has led to a 25 percent increase in the number of patients treated in secondary healthcare. The guiding principles for psychiatric healthcare are accessibility, quality, and patient-centered treatment, but a lack of resources can make it difficult to live up to these principles.

## Historical view

In Denmark, psychiatry has been a hot topic in the public debate for many years. The previous government (2019 to 2022) decided to initiate a 10-year plan for psychiatry. The content of the plan was developed in collaboration between the Danish Health Authority and the Danish Authority for Social Services and Housing, and it involved many stakeholders [2].

In November 2022, the newly elected government published its policy for its time in office. In it was stated that a substantial investment would be made in the 10-year plan for psychiatry. The government planned to increase the annual expenses by 3 billion Danish krones (DKK), equivalent to 400 million Euros. Since 2019, 1,1 billion DKK have already been allocated for psychiatry annually. Taken together, these investments are equivalent to an approximately 18% increase in the total budget for psychiatry in Danish regions and municipalities.

We are still awaiting the actual investment, and it is therefore too early to get a clear picture of what exact investments are planned. The specific areas of investment are not defined yet, and the release of the majority of funds is awaited.

A range of different initiatives has led to this remarkable decision regarding investments.

## Uniting all forces, NGOs, and professional organizations

An important reason for this positive development has been that NGOs and different professional groups have joined forces and are now united in an organization called the “Psychiatry Alliance.” The alliance includes all user organizations, overarching user and family organizations and disease-specific groups; and trade unions for doctors, nurses, social workers, occupational therapists, physiotherapists, pedagogues, and nurse aids; and scientific societies for psychiatrists and psychologists. It has been a long process to reach this unification. At first, rather few organizations agreed on a limited number of common recommendations, but in September 2021, 45 organizations agreed on a document called “Psykiatriløftet,” which in Danish has a dual meaning as it means both “Promise to Psychiatry” and” Lifting Psychiatry.” It included eight recommendations covering key elements of importance for both user groups and professionals. This led to the organization’s support for the 10-year plan for psychiatry, developed by the national health and social authorities. The united effort has truly changed the picture, and we are so proud and happy to be part of a united force.

## Important data indicating a strong need for investment

In Denmark, like in other Nordic countries, we have extremely good access to register-based information. This has enabled us to provide important information in several areas. It was shown that mental illnesses represent 25 percent of the total burden of disease, and that it exceeds cancer (15 percent) and cardiovascular disease (17 percent) [3]. Data showing a 10–15-year reduction in life expectancy [4–6], as well as increased somatic comorbidity for people with mental disorders [7], and dramatically high suicide rates shortly after discharge from a psychiatric department (200–400 fold higher than the general population) [8] played an important role in the public debate. Several epidemiological studies showed that mental illnesses are major public health concerns. One-sixth of children in Denmark avail of mental health services before the age of 18 [9], and one-third of the population receive treatment from mental health services during their lifetime [10]. Finally, Danish figures regarding 15–20 years of lost contribution to the labor market were influential [11]. Together with the reports from the WHO about treatment gaps [12], these data gave us strong arguments for the need for better services for people with mental disorders. This information was also presented in a popular Danish book titled *How to create the psychiatry of the future* [13]. The book was one in a series of books, dealing with important societal problems. It presented the current status regarding inequality between somatic and psychiatric healthcare with regard to access to treatment, quality of treatment, excess mortality, and stigma. It also presented principles for future solutions.

**Figure:**
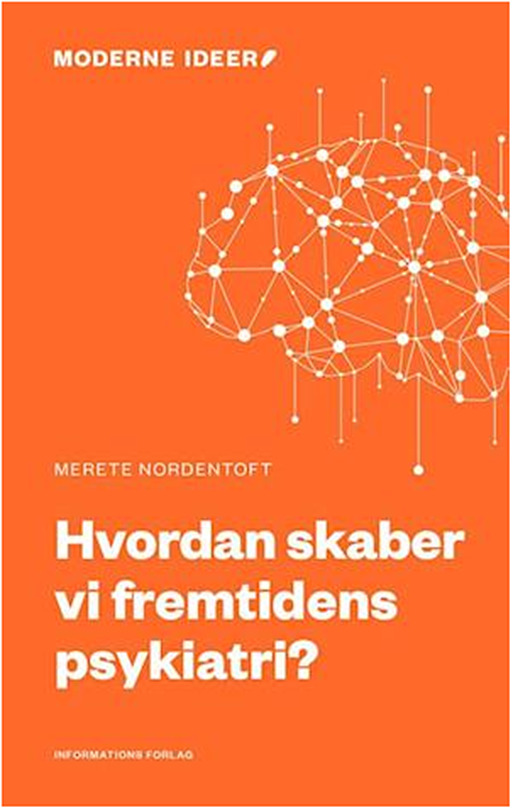

**Figure 1.**
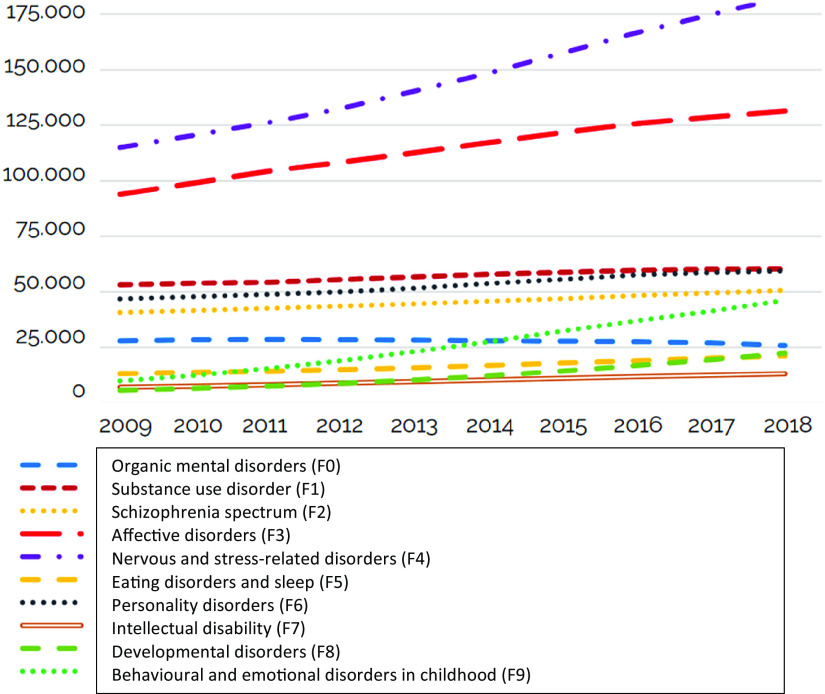
Time change in the number of adults (above 18 years old) receiving psychiatric treatment in the secondary health sector. *Source*: https://www.sst.dk/-/media/Udgivelser/2022/psykiatriplan/KORT_10AARS_PSYK-PLAN_100122_EN_11-maj.ashx [2].

Moreover, a substantial increase in people seeking help from psychiatric services (see Figure 1), and an increasing number of young people reporting failure to thrive [14] were of concern for healthcare providers, politicians, and the public alike. Another concern was that rates of readmissions within 30 days after discharge from the psychiatric department increased from 21 percent in 2014 to 24 percent in 2022.

Taken together, all this information served as strong arguments for the need for enhancement and improvement of psychiatric services.

## Collaboration with media

Many of the organizations in the Psychiatry Alliance had built good relationships with journalists in national television, radio, and large newspapers. We gave many interviews and appeared frequently in different media. All the largest organizations had employed full-time or part-time journalists, who helped to write chronicles and debate posts and made sure that these were published. Often several or all organizations in the Psychiatry Alliance wrote viewpoints together. Social media were used to disseminate the most crucial messages, and to emphasize that all organizations were agreeing with and supported each other.

Many journalists have a strong interest in the cause and took initiatives to ask the politicians relevant questions. In that way the media were really helpful in making psychiatry an important issue in the public debate and the upcoming election in October 2022. The media frequently reported the scientific literature, and we had a constructive collaboration, where the scientists explained the findings and the journalists tried to communicate it as a news story.

The main focus points we emphasized were that psychiatric illnesses are impacting all of us, the current health and social systems are not capable of delivering the necessary support, and a substantial improvement, for the patients and society as a whole, is possible with the right interventions.

During the general election, psychiatry ranked as the fourth most important issue for voters right after climate, economy, and general healthcare.

## National plan

Several consecutive governments have taken initiatives to increase investments in psychiatry. There have been investments in new and modern hospitals, and some rather modest increases in budgets for staff. However, in 2019, a newly elected Social Democratic government decided together with the supporting other parties that there should be a 10-year plan for psychiatry. At the same time, 600 million DKK (equivalent to 80 million Euros) were invested in psychiatry. The work with the 10-year plan was interrupted by the pandemic. After this unplanned delay, the plan ended up as a very strong document, which represented key points from all stakeholders and was supported by all user groups and professional organizations. In January 2022, the plan was launched by national health and social authorities [2]. It included a very harsh criticism of the current state of mental health services, and 37 recommendations for improvement and five main priorities were presented. After negotiations with different organizations and parties in the government, this plan was agreed upon by all parties in the parliament except one. At first, a rather small budget was decided (0.5 billion DKK equivalent 66 million Euros), and the user organizations called it Psychiatry Plan 1, expecting more plans to come.

After elections in October 2022, a new government was formed representing three large parties. In the government foundation, it was stated that the annual budget for psychiatry over a 10-year period would be increased by 4 billion DKK, equivalent to 0.53 billion Euros. This corresponds to an increase of approximately 18 percent in the total budget for mental healthcare in Danish regions and municipalities.

## Presenting calculation of budget

Already in November 2021, the Danish Psychiatric Society, the Danish Child and Adolescent society, and a professor in health economy (JK) took the initiative to calculate what we considered a realistic budget for what was necessary to meet the needs for psychiatric care in primary and secondary healthcare and in social services. This budget included 4.5 billion DKK (0.6 billion Euros) for annual running cost and another 3.5 billion for capital expenditure. We were the first to present a budget, and the figures were widely cited in the public debate. We were afraid that the budget for the announced 10-year plan would be rather small, so we took this initiative to set the bar for our expectations. It proved to be very consequential to the final budget.

## Assertive approach toward politicians

Several organizations in the Psychiatry Alliance had contact with politicians from the government, supporting parties, and the opposition. Our experience was that that psychiatry was considered an important area across the political spectrum, and that a substantial part of voters was represented by our organizations. We had frequent meetings and telephone and mail/text contact during the political process and negotiations. Substantial political influence can be gained this way, especially if conducted in a structured and united manner.

## Take-home message

The data supporting our arguments for a 10-year plan for psychiatry are based on high-quality Danish register-based analyses, but our findings are very likely to be representative of other countries, which has also been demonstrated in meta-analyses [15]. Overall, the good reasons for investments are generalizable. In the development of the Danish cancer plans, comparisons across countries played a pivotal role, as politicians did not want Denmark to have a worse outcome than other Nordic countries. This approach can be transferred to the field of psychiatry. Also, the 10-year plan for psychiatry was modeled after the cancer plans that had transformed the field over the last decades. This emphasizes the imperative of a comprehensive plan for handling mental illnesses in contrast to scattered initiatives, and highlights the need for an approach as effective as seen in oncology.

Our experiences with uniting forces can be brought to other countries, and we will strongly recommend psychiatrists to unify with organizations for families and users, as well as other professional groups. It takes a while, and there can be a lot of skepticism, but it can be solved. We also strongly recommend colleagues in other countries to be assertive toward politicians and media and to invite politicians to open large meetings about psychiatric themes. Being a first mover and presenting tentative budgets can be very influential.

## References

[1] Nordentoft M, Krantz MF, Hageman I. Right-based mental health care-advantages of tax-financed universal mental health care: lessons from Denmark. JAMA Psychiatry. 2022;79(1):7–8. 34730789 10.1001/jamapsychiatry.2021.3167

[2] Danish Health Authority. Strengthening mental health care. Recommendations for a 10-year action plan in Denmark. Copenhagen: Danish Health Authority; 2022.

[3] Sundhedsstyrelsen SIfF. [Sygdomsbyrden i Danmark], burden of disease in Denmark. Copenhagen: Sundhedsstyrelsen; 2015.

[4] Nordentoft M, Wahlbeck K, Hallgren J, Westman J, Osby U, Alinaghizadeh H, et al. Excess mortality, causes of death and life expectancy in 270,770 patients with recent onset of mental disorders in Denmark, Finland and Sweden. PLoS One. 2013;8(1):e55176. 23372832 10.1371/journal.pone.0055176 PMC3555866

[5] Wahlbeck K, Westman J, Nordentoft M, Gissler M, Laursen TM. Outcomes of Nordic mental health systems: life expectancy of patients with mental disorders. Br J Psychiatry. 2011;199(6):453–8. 21593516 10.1192/bjp.bp.110.085100

[6] Plana-Ripoll O, Pedersen CB, Agerbo E, Holtz Y, Erlangsen A, Canudas-Romo V, et al. A comprehensive analysis of mortality-related health metrics associated with mental disorders: a nationwide, register-based cohort study. Lancet. 2019;394(10211):1827–35. 31668728 10.1016/S0140-6736(19)32316-5

[7] Momen NC, Plana-Ripoll O, Agerbo E, Benros ME, Børglum AD, Christensen MK, et al. Association between mental disorders and subsequent medical conditions. N Engl J Med. 2020;382(18):1721–31. 32348643 10.1056/NEJMoa1915784 PMC7261506

[8] Madsen T, Erlangsen A, Hjorthoj C, Nordentoft M. High suicide rates during psychiatric inpatient stay and shortly after discharge. Acta Psychiatr Scand. 2020;142(5):355–65. 32715465 10.1111/acps.13221

[9] Dalsgaard S, Thorsteinsson E, Trabjerg BB, Schullehner J, Plana-Ripoll O, Brikell I, et al. Incidence rates and cumulative incidences of the full spectrum of diagnosed mental disorders in childhood and adolescence. JAMA Psychiatry. 2020;77(2):155–64. 31746968 10.1001/jamapsychiatry.2019.3523 PMC6902162

[10] Pedersen CB, Mors O, Bertelsen A, Waltoft BL, Agerbo E, McGrath JJ, et al. A comprehensive nationwide study of the incidence rate and lifetime risk for treated mental disorders. JAMA Psychiatry. 2014;71(5):573–81. 24806211 10.1001/jamapsychiatry.2014.16

[11] Plana-Ripoll O, Weye N, Knudsen AK, Hakulinen C, Madsen KB, Christensen MK, et al. The association between mental disorders and subsequent years of working life: a Danish population-based cohort study. Lancet Psychiatry. 2023;10(1):30–9. 36480953 10.1016/S2215-0366(22)00376-5

[12] Patel V, Saxena S, Lund C, Thornicroft G, Baingana F, Bolton P, et al. The lancet commission on global mental health and sustainable development. Lancet. 2018;392(10157):1553–98. 30314863 10.1016/S0140-6736(18)31612-X

[13] Nordentoft M. Fremtidens psykiatri (future of psychiatry). Copenhagen: Informations forlag; 2018.

[14] Sundhedsstyrelsen; SDU SIfF. Danskernes sundhed. Den nationale sundhedsprofil 2021. København; 2022.

[15] Correll CU, Solmi M, Croatto G, Schneider LK, Rohani-Montez SC, Fairley L, et al. Mortality in people with schizophrenia: a systematic review and meta-analysis of relative risk and aggravating or attenuating factors. World Psychiatry. 2022;21(2):248–71. 35524619 10.1002/wps.20994 PMC9077617

